# Sorbitol-Stabilized Silicon Formulation Improve Root Traits and Antioxidant Response in Drought-Stressed Soybean

**DOI:** 10.3390/plants15020197

**Published:** 2026-01-08

**Authors:** Felipe Sousa Franco, Jonas Pereira de Souza Júnior, Renato de Mello Prado, Milton Garcia Costa, Cid Naudi Silva Campos, Leonardo Motta Berzaghi Junior, Nícolas Leite Capucin, Gustavo Paparotto Lopes, Gabriel Sgarbiero Montanha, Marcia Leticia Monteiro Gomes, Ana Carina da Silva Cândido Seron, Hudson Wallace Pereira de Carvalho, José Lavres, Renan Caldas Umburanas

**Affiliations:** 1 Laboratory of Nuclear Instrumentation, Center of Nuclear Energy in Agriculture, University of São Paulo, 303 Centenário Avenue, Piracicaba 13416-000, SP, Brazil; f.sousa.franco@usp.br (F.S.F.);; 2 Sustainable Tropical Agriculture Center, Luiz de Queiroz College of Agriculture (ESALQ/USP), University of São Paulo, 11 Pádua Dias Avenue, Piracicaba 13416-900, SP, Brazil; 3 Citrus Research and Education Center, University of Florida, 700 Experiment Station Rd, Lake Alfred, FL 33850, USA; jpereiradesouza@ufl.edu; 4 School of Agricultural and Veterinarian Sciences, São Paulo State University (Unesp), Access Route Prof. Paulo Donato Castellane s/n, Jaboticabal 14884-900, SP, Brazil; 5 Campus de Chapadão do Sul (CPCS), Universidade Federal de Mato Grosso do Sul (UFMS), Chapadão do Sul 79560-000, MS, Brazil

**Keywords:** *Glycine max* (L.) Merril, water deficit, foliar application, flavonoids, Si

## Abstract

Silicon (Si) plays a critical role in regulating plant physiological processes, particularly through its influence on non-enzymatic antioxidant systems and amino acid metabolism. This study aims to assess soybean performance in response to both soil and foliar Si applications under well-watered and drought conditions, with the goal of enhancing Si accumulation in plant tissues and potentially strengthening the crop’s physiological responses to water deficit stress. This is especially pertinent given that the mechanisms underlying Si fertilization and its contribution to drought tolerance in soybean remain poorly understood. Greenhouse experiments were conducted using a 3 × 2 factorial design. The factors were: (i) three foliar Si treatments: control (no Si), potassium silicate (SiK; 128 g L^−1^ Si, 126.5 g L^−1^ K_2_O, pH 12.0), and sorbitol-stabilized potassium silicate (SiKe; 107 g L^−1^ Si, 28.4 g L^−1^ K_2_O, 100 mL L^−1^ sorbitol, pH 11.8); and (ii) two soil water levels: well-watered (80% field capacity) and water-restricted (40% field capacity), the latter simulating tropical dry spells. Silicon was applied to the soil via irrigation and to the leaves via foliar spraying prior to the onset water restriction. All Si solutions were adjusted to pH 7.0 with 1 M HCl immediately before application. Potassium (K) levels were standardized across treatments through supplementary applications of KCl to both soil and foliage. Biometric and physiological parameters were subsequently measured. Sorbitol-stabilized Si enhanced Si accumulation in soybean tissues and improved plant resilience under both well-watered and drought conditions by promoting key physiological traits, including increased levels of daidzein and ascorbic acid levels, along with reduced amino acid concentrations. It also improved biometric parameters such as leaf area, root development, and number of pods per plant. These findings further support the role of Si as a beneficial element in enhancing stress tolerance and contributing to sustainable agricultural practices.

## 1. Introduction

Climate change is increasing the frequence and intensity of extreme weather events. Prolonged heat waves and droughts, as well as excessive rainfall leading to floods, mudslide and erosion [1], are becoming increasingly common, particularly in tropical and subtropical regions such as Brazil, one of the world’s biggest agricultural producers [2]. Consequently, identifying practices that enhance plant resilience to extreme weather conditions is essential for ensuring economic-environmental sustainability and global food security.

Soybean (*Glycine max* (L.) Merril) is the fourth most cultivated crop globally and the leading agricultural crop in Brazil, where it covered 46 million hectares during the 2024/2025 season [3]. Its widespread cultivation, both in Brazil and worldwide, exposes it to a wide range of climatic conditions, particularly drought, since soybean is a drought-sensitive herbaceous species. Given the crop’s limited resilience to severe climate events, there is a clear need to enhance its capacity to withstand such stresses. Climate change is expected to exacerbate these challenges by concentrating rainfall and extending dry periods, further emphasizing the urgency of developing strategies to improve crop resilience and ensure food security [4]. An analysis of soybean production data in Brazil from 1970 to 2023 showed that in years marked by prolonged drought, yield losses reached up to 30% [5].

Silicon (Si), the second most abundant element in the earth crust (~28%) [6], is recognized as a beneficial element with potential to increase yield in various crops [7,8,9]. Silicon application has also been reported to attenuate both biotic stresses, such as pests and diseases, and abiotic stresses, including shading, drought, radiation, temperature extremes, salinity, toxic elements and nutrient imbalances [10,11,12]. However, the beneficial effects of Si depend on factors such as plant uptake efficiency, developmental stage, and Si availability in the soil [9,13,14,15,16]. Additionally, the composition of Si-containing solutions, particularly the inclusion of sorbitol, may enhance their effects on both physiological processes and structural development in soybean plants, thereby improving their ability to withstand drought stress.

Drought stress occurs when the rate of water transpiration to the atmosphere exceeds the amount of water that plant roots can absorb from the soil [17,18]. Water deficiency reduces cell division rate, cell elongation and differentiation, impairs the leaves thermal regulation, and increases damage to cell membranes, leading to the accumulation of reactive oxygen species (ROS) and reactive nitrogen species (RSN). It can also degrade chlorophyll pigments, reduce photosynthetic activity and metabolite production, ultimately lowering yield potential [11,19,20].

Under normal conditions, cells maintain a balance between ROS production and scavenging. However, abiotic or biotic stress disrupts this equilibrium, resulting in excessive ROS accumulation and oxidative damage [21]. Silicon has been shown to regulate cell wall structure, maintaining membrane integrity, modulate carbohydrate metabolism, and promote proline accumulation [22]. Proline reduces the toxic effects of ROS [10], and stabilizes signaling proteins involved in drought tolerance, such as kinases and transcription factors [10]. In common bean (*Phaseolus vulgaris* L.), Si application has been reported to increase osmolyte and metabolite accumulation, thereby enhancing cell turgor and water uptake [23]. Silicon also stimulates polyamine and 1-aminocyclochloropane-1-carboxylic acid (ACC) expression, promotes root development, and increases the root surface area available for water absorption [13].

Furthermore, gene expression analyses have shown that Si application upregulates the expression of enzymes involved in lignin biosynthesis in the stem, thereby reducing the risk of lodging [12]. A reduction in leaf area and an increase in leaf thickness has also been reported following foliar Si application, without affecting chlorophyl accumulation under either full sunlight or shading conditions [12]. In soybean exposed to both water deficit and UV-B radiation, Si application (1.7 mM) was found to increase stomatal conductance, reduce the accumulation of anthocyanins and phenolic compounds, enhance antioxidant enzyme activity, and promote plant growth [14].

The benefits of Si to soybeans have also been attributed to the polymerization of Si beneath the epidermis, which helps reduce excessive transpiration [22,24,25,26]. Silicon supplementation has been shown to alleviate salt stress in soybean by enhancing the production of non-enzymatic antioxidants, such as phenolic compounds and ascorbic acid [27].

Leaf Si absorption can be enhanced by combining it with sorbitol, which lowers the solution’s deliquescence point, reduces droplet evaporation, and facilitates uptake [24]. This improves spray stability and, when combined with Si, can enhance the absorption in soybean, maize and cotton [24]. The central question of this study is how a sorbitol-stabilized Si solution affects various physiological and biometric traits in soybean under both well-watered and drought conditions. Although numerous studies have demonstrated the benefits of Si in enhancing plant tolerance to abiotic stresses, the underlying physiological and metabolic mechanisms, particularly in soybeans, remain insufficiently understood. In this context, the objective of this study is to evaluate how increasing Si content in plant tissues, especially through a sorbitol-stabilized formulation, influences the physiological, biochemical, and biometric traits of soybean under both well-watered and drought conditions. We hypothesize that Si mitigates drought stress, enhances agronomic performance, and stimulates the production of osmolytes and non-enzymatic antioxidants in soybean cells—effects that are expected to be more pronounced when a stabilized Si source is used.

## 2. Results

### 2.1. Experiment I

**Figure 1:**
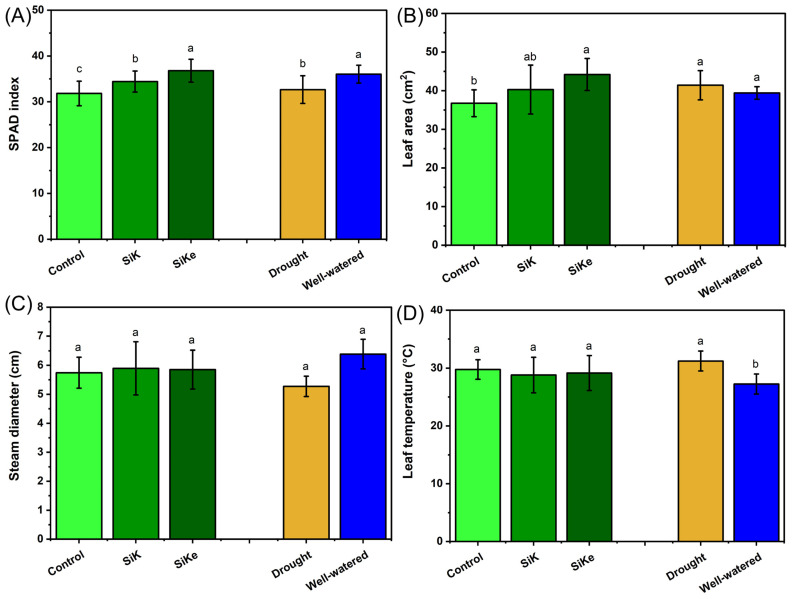
(**A**) SPAD index; (**B**) Leaf area; (**C**) Stem diameter; (**D**) Leaf temperature. Lowercase letters indicate significant differences between the control (with no Si application), potassium silicate (SiK), and potassium silicate stabilized with sorbitol (SiKe) treatments. Significance was determined at Tukey’s HSD (α = 0.05). Detailed *p*-values for each factor can be found in Supplementary Material Table S3.

For the SPAD index (Figure 1A), the potassium silicate stabilized with sorbitol (SiKe) treatment resulted in higher values, regardless of water availability, 6.8% and 15.5% higher than potassium silicate (SiK) and the control (no Si application), respectively. Additionally, well-watered plants exhibited a 10.2% higher SPAD index compared to those under drought conditions. Leaf area (Figure 1B) showed the greatest response under the SiKe treatment, with increases of 9.6% and 20.3% compared to SiK and the control, respectively, regardless of water status. Stem diameter (Figure 1C) did not differ among the Si treatments; however, drought stress reduced stem diameter by 21% compared to well-watered plants. Leaf temperature (Figure 1D) was not influenced by Si treatment, but increased 14.6% under drought conditions relative to well-watered conditions.

**Figure 2:**
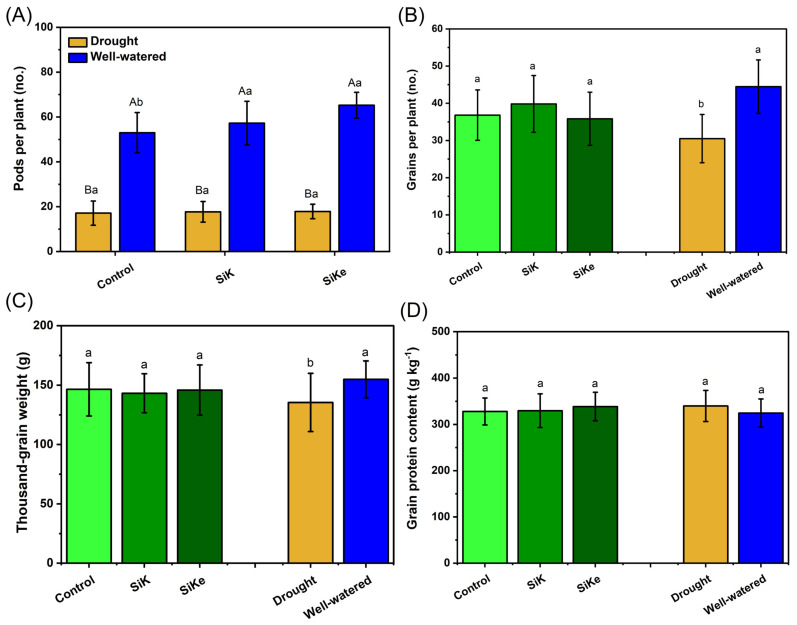
(**A**) Pods per plant; (**B**) Grains per plant; (**C**) Thousand-grain weight; (**D**) Grain protein content. Lowercase letters indicate significant differences between the control (with no Si application), potassium silicate (SiK), and potassium silicate stabilized with sorbitol (SiKe); uppercase letters compare the water conditions (drought and well-watered) within each Control, SiK, and SiKe treatments. Significance was determined at Tukey’s HSD (α = 0.05). Detailed *p*-values for each factor can be found in Supplementary Material Table S3.

The number of pods per plant (Figure 2A) did not differ among the Si treatments under drought conditions. However, under well-watered conditions, pod number increased by 8.0% and 23.1% compared to the control in SiK and SiKe treatments, respectively. The number of grains per plant (Figure 2B) also showed no significant differences among the Si treatments. Nevertheless, well-watered plants produced 45.8% more grain per plant than those under drought-stressed conditions. For thousand-grain weight (Figure 2C), no differences were observed among the Si treatments. However, well-watered conditions resulted in a 14.4% increase compared to drought-stressed plants. Grain protein content (Figure 2D) showed no differences among treatments.

### 2.2. Experiment II

**Figure 3:**
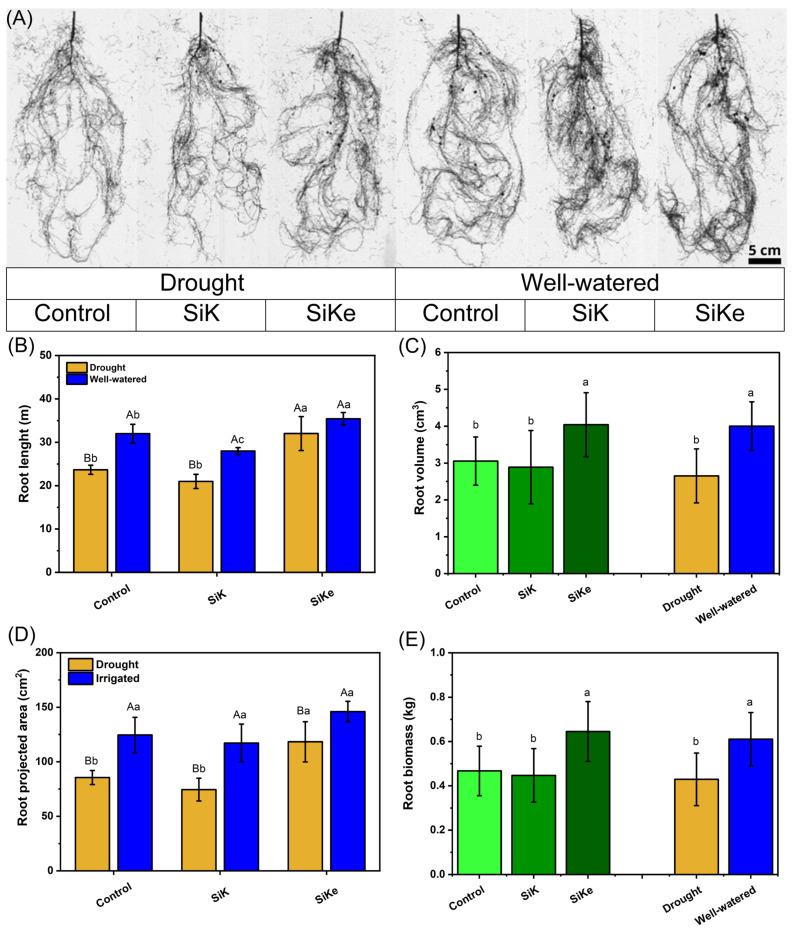
(**A**) Samples of images for the evaluation of root attributes; (**B**) Root length; (**C**) Root volume; (**D**) Root projected area; (**E**) Root biomass. The black dots represent nodules formed on the roots. Lowercase letters indicate significant differences between the control (with no Si application), potassium silicate (SiK), and potassium silicate stabilized with sorbitol (SiKe) treatments; uppercase letters compare the water conditions (drought and well-watered) within each Control, SiK, and SiKe treatments. Significance was determined at Tukey’s HSD (α = 0.05). Detailed *p*-values for each factor can be found in Supplementary Material Table S3.

In Figure 3A illustrates the effects of Si treatments and water availability on the root structure and development of soybean plants, based on images collected during the analysis of root attributes. Root length exhibited a significative interaction between Si treatment and water availability (Figure 3B). Under drought stress, the SiKe treatment increased root length by 52.6% and 35.3% compared to SiK and the control, respectively. Under well-watered conditions, all Si treatments differed, with SiKe producing the longest roots, 26.6% and 10.7% longer than SiK and control, respectively.

Root volume differed among Si treatments and water availability levels (Figure 3C). The SiKe treatment increased root volume by 44.4% and 38.1% compared to SiK and the control, respectively, regardless of water status. Additionally, well-watered conditions increased root volume by 42.3% compared to drought conditions.

A significant interaction between Si and water availability as observed for root projection area (Figure 3D). Under drought conditions, the SiKe treatment increased root projection area by 96.3% and 70.9% compared to SiK and the control, respectively. Under well-watered conditions, no differences were observed among treatments. Root biomass increased 44.4% and 38.1% in the SiKe treatment compared to SiK and the control, repectively (Figure 3E). In relation to water availability, well-watered conditions increased root biomass by 45.2% compared to drought-stressed plants.

**Figure 4:**
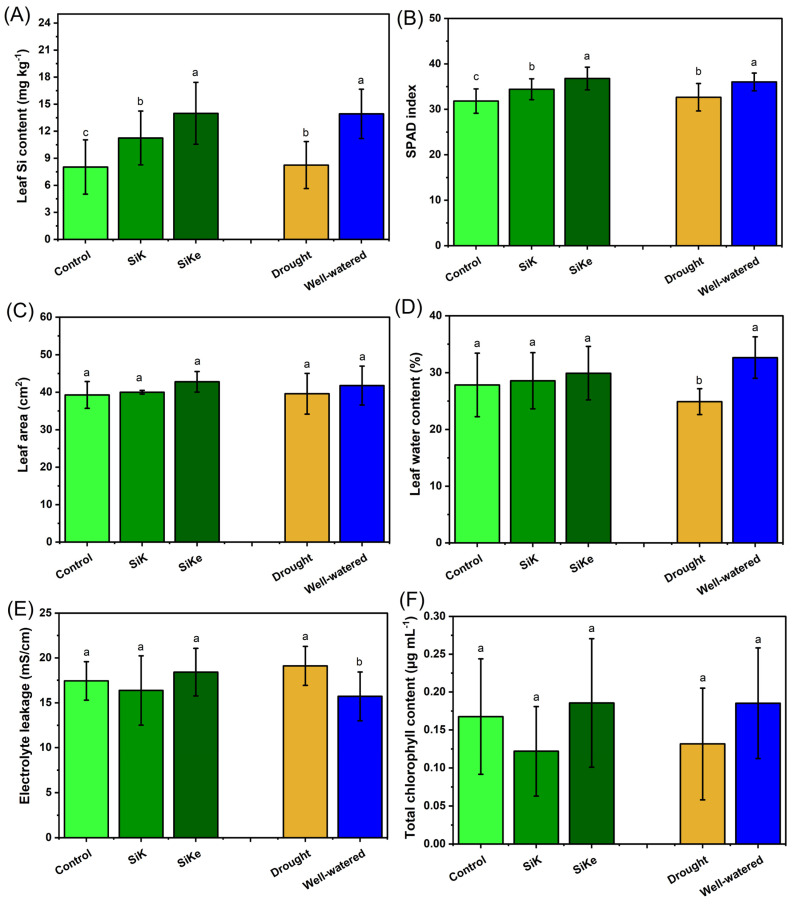

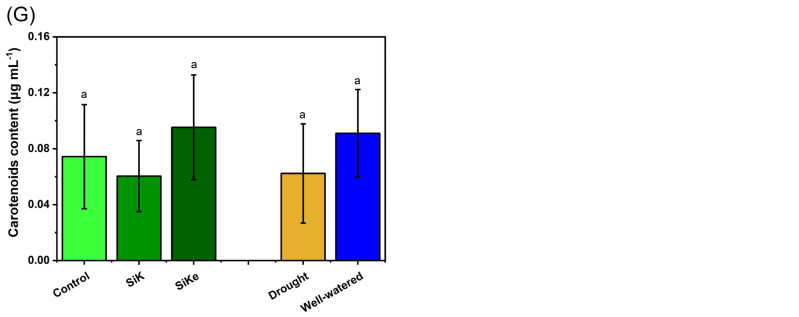
(**A**) Leaf Si content; (**B**) SPAD index; (**C**) Leaf area; (**D**) Leaf water content; (**E**) Electrolyte leakage; (**F**) Total chlorophyll content; and (**G**) Carotenoids content. Lowercase letters indicate significant differences between the control (with no Si application), potassium silicate (SiK), and potassium silicate stabilized with sorbitol (SiKe) treatments. Significance was determined at Tukey’s HSD (α = 0.05). Detailed *p*-values for each factor can be found in Supplementary Material Table S3.

Leaf Si content in the SiKe treatment was 24.2% and 74.7% higher than in SiK and the control, respectively (Figure 4A). Under well-watered conditions, leaf Si content was 69.7% higher compared to drought-stressed plants. No differences were observed in the SPAD index among Si treatments or between water conditions (Figure 4B). Similarly, leaf area did not differ with respect to Si treatment or water availability (Figure 4C), possibly because measurements were taken at the R_3_ phenological stage.

Leaf water content was not affected by Si treatments but was significantly influenced by water condition (Figure 4D), with well-watered plants showing a 31.1% higher water content compared to drought-stressed plants. Electrolyte leakage (Figure 4E) was also unaffected by Si treatments; however, it increased by 21.6% under drought conditions relative to well-watered conditions. Chlorophyll content (Figure 4F) did not differ among Si treatments or between water availability levels, and a similar trend was observed for carotenoid content (Figure 4G).

**Figure 5:**
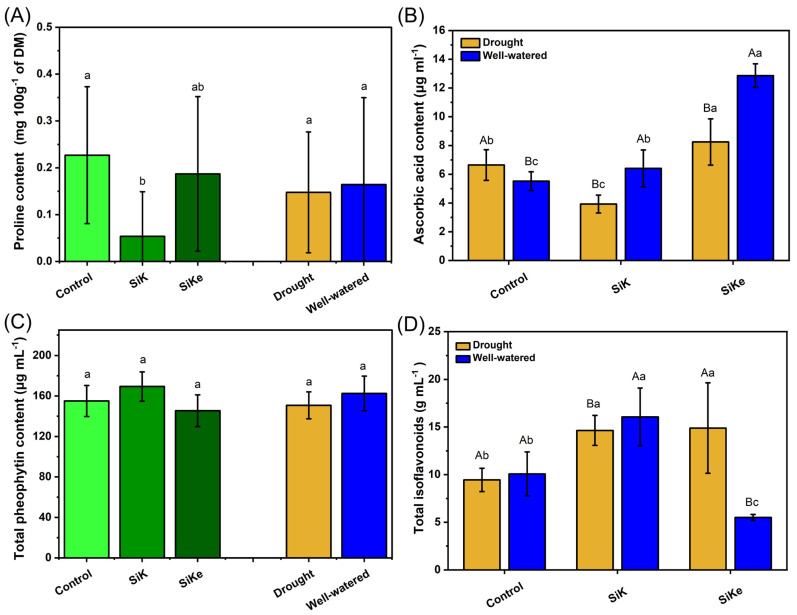
(**A**) Proline content; (**B**) Ascorbic acid content; (**C**) Total pheophytin content; (**D**) Total isoflavonoids. Lowercase letters indicate significant differences between the control (with no Si application), potassium silicate (SiK), and potassium silicate stabilized with sorbitol (SiKe) treatments; uppercase letters compare the water conditions (drought and well-watered) within each Control, SiK, and SiKe treatments. Significance was determined at Tukey’s HSD (α = 0.05). Detailed *p*-values for each factor can be found in Supplementary Material Table S3.

Proline content in the control treatment was 326% higher than SiK, but did not differ from SiKe. Water availability had no effect on proline levels (Figure 5A). Ascorbic acid content showed a significant interaction between Si treatments and water availability (Figure 5B). Under drought conditions, ascorbic acid levels in the SiKe treatment were 110% and 24% higher than in SiK and the control, respectively. Under well-watered conditions, Si treatments also differed, with SiKe resulting in ascorbic acid levels that were 100% and 132% higher than those observed in SiK and the control, respectively.

Total pheophytin content did not differ between Si treatments or between water availability (Figure 5C). For total isoflavonoids content, an interaction was observed between Si treatment and water availability (Figure 5D). Under drought conditions, both SiKe and SiK increased isoflavonoid levels by 58.2% and 55.0%, respectively, compared to the control. Under well-watered conditions, SiK showed the highest response, with isoflavonoid content 59.3% and 191% higher than in the control and SiKe treatments, respectively.

**Figure 6:**
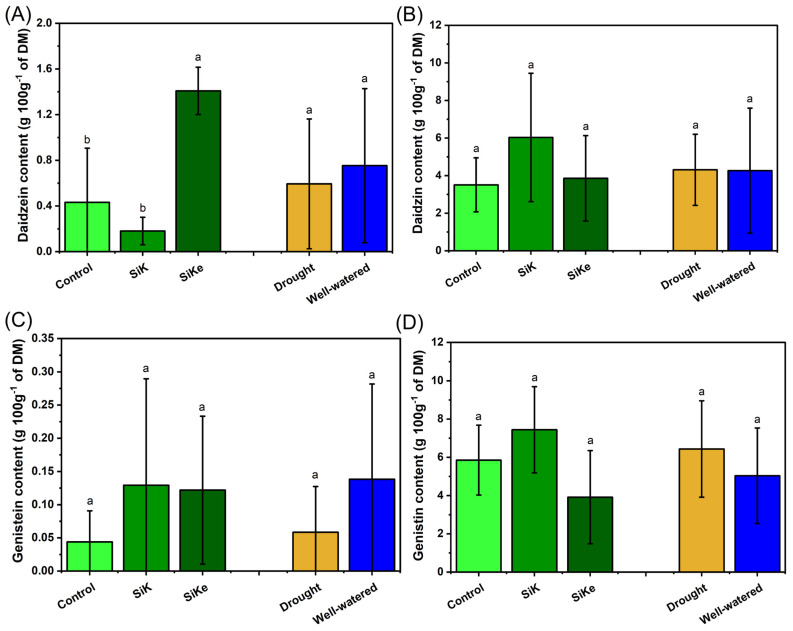
(**A**) Daidzein content; (**B**) Daidzin content; (**C**) Genistein content; (**D**) Genistin content. Lowercase letters indicate significant differences between the control (with no Si application), potassium silicate (SiK), and potassium silicate stabilized with sorbitol (SiKe) treatments. Significance was determined at Tukey’s HSD (α = 0.05). Detailed *p*-values for each factor can be found in Supplementary Material Table S3.

For daidzein content, only the Si treatment had a significant effect (Figure 6A). The SiKe treatment increased daidzein levels by 677% compared to SiK and by 226% compared to the Control. No differences were observed in response to the water condition. For Daidzin, Genistein, and Genistin, no effects were detected from either the Si treatments or water conditions (Figure 6B–D).

### 2.3. Principal Components Analysis (PCA)

**Figure 7:**
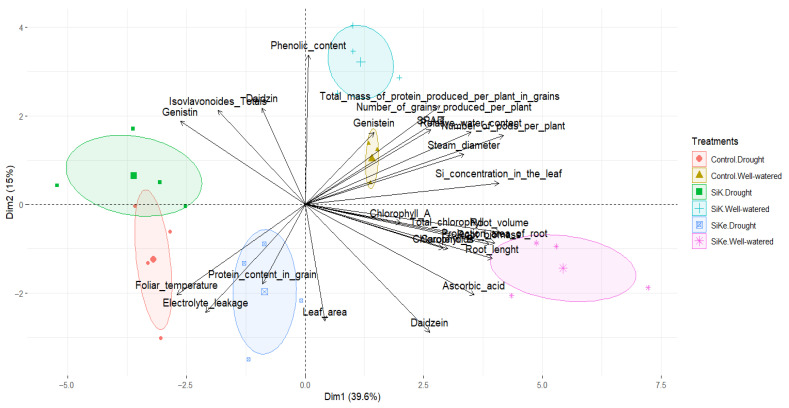
Principal Component Analysis (PCA) of all variables presented as graphs in this article, showing the distribution of factors according to PC1 and PC2. The analysis was performed in RStudio environment. To detail the contribution of each variable to PC1 and PC2, we have included the full loadings in Table S4 of the Supplementary Material.

Based on the data from Experiments I and II the principal component analysis (PCA) revealed that the first two principal components (Dim1 and Dim2) explained 39.6% and 15% of the total variance, respectively (Figure 7), indicating that these components captured a substantial portion of the dataset’s structure. The PCA showed a clear separation between treatments, with well-watered plants clustering on the right side of the plot and drought-stressed plants on the left. Notably, stress-related variables, such as foliar temperature, electrolyte leakage, genistin, total isoflavonoids, and daidzin, were closely associated with drought treatments, reinforcing their potential as reliable indicators of stress response.

The SiKe treatment under well-watered conditions exhibited a strong association with root biometric traits, chlorophyll content, ascorbic acid, and daidzein, suggesting that sorbitol-stabilized Si positively influences root development and physiological parameters, thereby enhancing plant resilience. Additionally, variables such as leaf Si concentration, total grain protein per plant, SPAD index, number of pods per plant, genistein content, and stem diameter showed intermediate trends between the SiK and SiKe treatments. These results indicate that Si, particularly in its stabilized form, may promote physiological resilience even under non-stress conditions, potentially improving overall soybean performance.

### 2.4. Heatmap of Amino Acids Content in the Leaf

**Figure 8:**
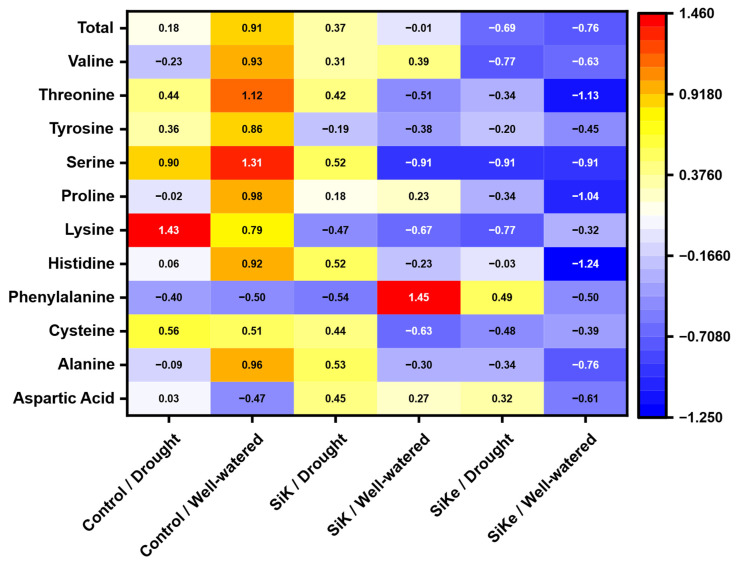
The heatmap shows Z-score correlations of amino acid contents (Aspartic acid, Alanine, Cysteine, Phenylalanine, Histidine, Lysine, Proline, Serine, Tyrosine, Threonine, Valine, and Total) with the factors control (with no Si application), potassium silicate (SiK), and potassium silicate stabilized with sorbitol (SiKe) under drought and well-watered conditions. The color gradients represent correlation intensity: red indicates Z-scores above the mean (positive correlations), yellow indicates values around the mean (moderate correlations), and blue indicates Z-scores below the mean (negative correlations). Statistical details, including *p*-values for each factor and the coefficient of variation (CV) for each amino acid, are provided in the Supplementary Material (Table S3).

High variability in leaf amino acid content was observed in the Experiment II, particularly among the control, SiKe, and SiK treatments, regardless of water availability (Figure 8). In the control treatment (both drought and well-watered conditions), the concentrations of several amino acids, notably threonine, serine, and lysine, were higher compared to SiK and SiKe. This pattern suggests a possible physiological modulation induced by Si, likely through the remobilization of these amino acids, thereby reducing their accumulation in plant tissues. Such an effect may contribute to improved metabolic homeostasis and, consequently, enhanced physiological efficiency in the plant.

Under well-watered conditions, control plants exhibited consistently higher z-scores for total amino acids, valine, threonine, serine, proline, histidine, and alanine compared to SiKe-treated plants, indicating a clear reduction in the relative accumulation of these metabolites in the SiKe/Well-watered treatment. Specifically, total amino acid content shifted from a strongly positive z-score in the Control/Well-watered group (0.91) to a markedly negative value in the SiKe/Well-watered group (−0.76). Valine followed a similar trend, decreasing from 0.93 to −0.63, while threonine exhibited an even more pronounced reduction, dropping from 1.12 to −1.13. Serine showed one of the most significant contrasts, declining from 1.31 to −0.91, and proline decreased from 0.98 to −1.04, indicating substantial suppression. Histidine presented the most intense decline among the amino acids analyzed, decreasing from 0.92 to −1.24, while alanine also dropped significantly, from 0.96 to −0.76. Collectively, these results suggest that SiKe application under non-limiting water conditions modulates amino acid metabolism. This likely reflects adjustments in nitrogen assimilation and carbon–nitrogen balance, rather than a stress-induced accumulation pattern.

Under drought conditions, the reduction in amino acid levels in SiKe-treated plants compared to the control was less pronounced than that observed under well-watered conditions. Nevertheless, the pattern still clearly demonstrated the metabolic modulation exerted by Si on plant physiology. This suggests that Si application influences amino acid metabolism even under water deficit, albeit with lower magnitude. Notably, the SiK treatment consistently showed intermediate responses between the control and SiKe, indicating a transitional metabolic adjustment influenced by the Si source and its interaction with drought stress.

## 3. Discussion

Over recent years, the use of Si has emerged as a promising strategy to mitigate the adverse effects of drought stress on plant development. The combination of Si with sorbitol has also been explored in previous study [24], showing positive effects on both biometric and physiological traits. Sorbitol enhances the stability of polar ligands networks in aqueous solutions, delaying the polymerization and precipitation of Si particle. Furthermore, it modifies the deliquescence point of salts, allowing for extended absorption of Si through the leaf surface [24].

In Experiment I, water deficit negatively affected the control treatment, despite the use of KCl to balance potassium levels in the SiK and SiKe sources. In contrast, SPAD index and leaf area were improved by both Si sources, regardless of water availability. These results suggest that Si enrichment may enhance tolerance to chlorophyll oxidative stress and improve water-use efficiency by sustaining cell division under stress conditions [28]. These findings are consistent with the study by Sah et al. [28], who observed increased leaf area in silicon-treated plants, particularly under high water availability. Similarly, Abdullah et al. [29] reported a 23% increase in photosynthetic rate in drought-stressed plants treated with 200 kg ha^−1^ of Si applied to the soil compared to the control. This improvement may be linked to the activation of the phenylpropanoid pathway and stress-responsive enzymes triggered by Si, which reduce the physiological impact of abiotic stress [30].

As expected, drought stress reduced stem diameter and increased leaf temperature. Water deficit is known to disrupt xylem hydraulic continuity by promoting cavitation and the formation of air embolisms, which impair water transport efficiency. Since transpiration-driven water flow is essential for heat dissipation and temperature regulation in leaves, reduced stomatal conductance under drought conditions further limits evaporative cooling. As a result, leaf temperature rises, and metabolic activity becomes restricted, as many enzymatic processes operate within narrow thermal optima. Regarding yield-related traits such as grain number per plant, thousand-grain weight, and grain protein content (Figure 2), no differences were observed among Si sources. This suggests that the drought stress occurring in the later stages of the crop cycle may have been too severe for Si to mitigate its effects, contrasting with previous reports of yield improvements under Si supplementation [30]. However, the number of pods per plant increased under irrigated conditions in both Si-treated groups (Figure 2), potentially due to improved flower retention during vegetative and early reproductive stages.

In Experiment II, root traits were improved by the application of sorbitol-stabilized Si. Both root volume and root biomass (Figure 3) increased under SiKe treatment, regardless of water availability. For root length and root projection area (Figure 3), an interaction between Si treatment and water condition was observed: under drought, SiKe promoted greater root growth compared to SiK and the control. These findings align with previous studies [13,29,31], which suggest that enhanced root development under Si supply may be linked to the modulation of gene expression associated with root growth—particularly genes involved in auxin biosynthesis and transport, such as YUC, PIN, and WAT. In addition, improved allocation of photoassimilates to belowground organs have been proposed as a complementary mechanism. Supporting this, Tripathi et al. (2022) [32] reported that soil-applied Si in soybean enhances the activity of phenolic compounds, osmoprotectants, and antioxidant enzymes. These biochemical responses help maintain chlorophyll homeostasis during drought stress and ensure a steady supply of sugars to support sustained root growth. Collectively, such mechanisms contribute to increasing plant resilience under adverse environmental conditions.

In the Experiment II, no consistent effects of Si treatments were observed on shoot and physiological traits such as leaf area, leaf water content, electrolyte leakage, total chlorophyll, carotenoids, SPAD index, or pheophytin content (Figure 3 and Figure 4). This variability may be attributed to environmental fluctuations during sampling period, which may have limited the detection of statistical differences, although SiKe generally showed a trend toward positive responses. Water deficit remained the predominant factor influencing these parameters, as shown in Table S3 of the Supplementary Material. In contrast, ascorbic acid content (Figure 5) showed a marked increase in response to SiKe treatment under both drought and well-watered conditions. Previous studies have reported that Si can stimulate ascorbic acid accumulation by enhancing the ascorbate–glutathione cycle, promoting reactive oxygen species (ROS) detoxification, and modulating metabolic pathways that support antioxidant biosynthesis [10].

Isoflavonoids analysis (daidzein, daidzin, genistein, and genistin) in Experiment II revealed that Si supply modulated secondary metabolism (Figure 6). Total isoflavonoid content exhibited an interaction between Si sources and water availability, with both SiK and SiKe treatments outperforming the control under both water conditions, except for SiKe under well-watered conditions, where the effect was less pronounced (Figure 5). These results suggest that Si may act as an elicitor of key genes and transcription factors involved in isoflavonoid biosynthesis. This is supported by findings from Chen et al. (2023) [33], Moreover, Si has been reported to enhance the activity of phenylalanine ammonia-lyase (PAL), a key enzyme in the phenylpropanoid pathway, thereby stimulating isoflavonoid synthesis [32,33]. This is consistent with a priming effect, in which Si promotes the basal accumulation of defense-related metabolites even under non-stressed conditions, contributing to redox homeostasis and enhancing physiological defense mechanisms [34,35]. Among the individual compounds analyzed, daidzein content showed a consistent increase in response to SiKe, regardless of water availability. This highlights Si’s role in modulating daidzein, a key molecule in plant defense and ROS scavenging [36].

Principal component analysis (PCA) further supported the experimental findings by revealing a clear separation between treatments based on water availability and Si application. Well-watered treatments (Control, SiK, and SiKe) formed a distinct cluster, reflecting differential physiological and biometric responses compared to drought-stressed treatments. Within the well-watered group, the SiKe treatment was strongly associated with vegetative growth parameters, including root length, root volume, plant height, and biomass, suggesting that SiKe promotes enhanced growth when water is not limited. In contrast, drought-associated traits such as increased leaf temperature, electrolyte leakage, and the accumulation of isoflavonoids (daidzein, genistein, and genistin) were more closely aligned with drought-stressed treatments, indicating characteristic metabolic responses to water restriction. Notably, some Si-treated plants under drought conditions displayed intermediate PCA profiles, suggesting a partial mitigating of drought effects. This may reflect the influence of Si on maintaining physiological and metabolic functions under stress, which appears to vary depending on Si accumulation in leaf tissues. Overall, PCA highlights the integrated influence of both water availability and Si supplementation on soybean physiology, growth, and metabolism, supporting the hypothesis that sorbitol-stabilized Si can enhance stress tolerance and performance through coordinated physiological and biochemical adjustments.

The heatmap analysis (Z-score) revealed pronounced heterogeneity in amino acid accumulation among treatments, depending on both water availability and Si application. Control plants, irrespective of water availability, accumulated higher levels of amino acid, which may represent an adaptive strategy to maintain osmotic potential under stress. In contrast, Si treated plants, particularly those receiving SiKe, exhibited lower amino acid levels, suggesting a shift in N allocation and metabolic regulation. As shown in Table S3 of Supplementary Material, the Si factor significantly affected the accumulation of specific amino acids, including histidine, lysine, serine, threonine and total amino acids. These compounds are known to play roles in osmoprotection and metabolic signaling, and their modulation likely contributes to a more balanced metabolic state in Si-treated plants.

Interestingly, in the context of drought stress, the observed patterns of proline accumulation suggest that adequate Si supply, particularly in the sorbitol-stabilized form (SiKe), reduced the accumulation of this amino acid, which is commonly recognized as a biochemical marker of water deficit. Si supplementation likely alleviated the perceived stress intensity, enabling plants to maintain metabolic homeostasis. This stress moderation may have resolved an energy allocation trade-off by negating the need for the energetically costly synthesis of osmoprotectants such as proline. As a result, limited carbon and nitrogen resources could be preferentially directed toward vegetative or reproductive growth processes [37]. Additionally, reduced proline levels may reflect a metabolic reallocation, in which this amino acid is diverted toward the biosynthesis of other stress-related compounds that support cellular homeostasis [37]. Such a shift is consistent with the increased level of isoflavonoids and ascorbic acid observed in the present study, suggesting a broader remobilization of nitrogenous metabolites toward antioxidant and defense pathways. The enhanced foliar absorption of SiKe, facilitated by sorbitol, likely contributed to this reprogramming of metabolic priorities.

Taken together, these results validate the hypothesis that Si supplementation can mitigate drought-induced stress in soybeans, stimulate antioxidant production, and modulate both primary and secondary metabolic pathways. Furthermore, the sorbitol-stabilized formulation (SiKe) consistently provided superior agronomic and physiological responses, indicating its potential as a more effective foliar Si fertilizer under both well-watered and drought conditions.

## 4. Materials and Methods

### 4.1. Pot Experiment and Location

The study was conducted in a greenhouse located in Piracicaba, SP, Brazil. Pots of 5 dm^3^ were used, and the soil employed was classified as a Typic Dystrophic Red Latosol [38]. Prior to the experiment, a chemical analysis of the soil was performed according to the methodology described by Raij et al. [39] (Table 1). The analysis revealed a Si content below 9.7 mg dm^−3^, indicating potentially low availability [40]. Soils with less than 20 mg dm^−3^ of available Si are considered deficient for Si-accumulating crops. Although soybean is not classified as a typical Si accumulator, this low availability may still limit potential Si physiological responses.

Two independent experiments were carried out. Experiment I was sown on 9 December 2023, at coordinates 22°42′ S, 47°37′ W, and altitude of 538 m, while experiment II was sown on 12 February 2024, at the same coordinates. The full schedule and timeline for both experiments are provided in the Supplementary Material (Table S1 and Table S2, respectively).

The soybean cultivar Dagma 6822 IPRO (Dagma Sementes Ltda., Dourados, MS, Brazil) was used in both experiments. Seeds were inoculated with 2 mL kg^−1^ of a commercial microbial solution Dual Force^®^ (Stoller ltda., Cosmópolis, SP, Brazil) containing *Bradyrhizobium japonicum* and *Azospirillum brasilense* (5 × 10^9^ CFU ml^−1^). Five seeds were sown per pot, and thinning was performed two weeks after emergence to maintain three plants per pot. No fertilizer correction was applied. The soil water retention capacity was determined to be 546 mL pot^−1^.

### 4.2. Experiment Design

The experiment was arranged in a 3 × 2 factorial design with five replicates. The first factor consisted of three Si sources: control (no Si application), potassium silicate (SiK) and sorbitol-stabilized potassium silicate (SiKe), the latter comprising SiK combined with Sorbitol [C_6_H_14_O_6_]. The second factor corresponded to water availability imposed after flowering stage (R_1_) [41], with two levels: 40% and 80% of the soil’s water retention capacity, representing drought and well-watered conditions, respectively. These levels were defined based on physiological thresholds, where 80% was identified as optimal for soybean development, and 40% represented the point at which visible wilting symptoms were observed [42,43].

Foliar applications of Si were carried out at three growth stages: V3 [44] (two fully developed trifoliate leaves), V5 (four fully developed trifoliate leaves), and R1 (beginning of flowering). Applications were conducted in the early morning (6:00 a.m.) under controlled environmental conditions, with relative humidity of 80% ± 10% and temperature of 20 °C ± 5%, monitored using an Elitech^®^ datalogger (San Jose, CA, USA). A portable CO_2_-pressurized sprayer (2.0 bar), equipped with fan-type nozzles, (Jacto^®^, model JGT 11002, Pompéia, SP, Brazil) was used. The sprayer was calibrated to a flow rate of 1.5 L min^−1^ per nozzle, simulating a spray volume of 150 L ha^−1^.

For each foliar application, a volume of 3.91 mL per plant was, applied, corresponding to 9.48 mmol L^−1^ of Si and 18.96 mmol L^−1^ of K for all treatments. In SiKe formulation, 2.03 mmol L^−1^ of sorbitol (70%; Quimisulsc^®^ Comércio de Produtos Químicos Ltda., São Paulo, SP, Brazil) was also included in the solution. The solutions were prepared immediately prior to application, and the pH was adjusted to 7.0 using 1 M HCl. The adjuvant FluiFlex^®^ (Agrichem, Ribeirão Preto, SP, Brazil) was added at 0.1% *v*/*v* to improve foliar absorption, and was also included in the control, which received only KCl to ensures equivalent potassium supply.

Soil applications of Si were performed daily over an 80-day period, from sowing until the onset of water deficit phase. A volume of 200 mL per pot of solution was applied, containing 2.8 mmol L^−1^ of Si and 5.6 mmol L^−1^ of K, with the pH adjusted to 7.0 using 1 M HCl. This concentration was selected to avoid Si polymerization process, a phenomenon that typically occurs at concentrations above 3 mmol L^−1^, reducing the efficacy of Si uptake [45]. In the control treatment, equivalent amounts of K were supplied via both foliar and soil applications using potassium chloride (KCl) as the K source.

Throughout the experiment, soil moisture was monitored daily by weighing the pots to ensure consistency in water availability. The drought treatment was initiated at the R_1_ phenological stage in both experiments and maintained for 20 days (Tables S1 and S2 of Supplementary Material).

### 4.3. Experiment I Measurements

The SPAD index, leaf area, stem diameter, and leaf temperature were evaluated at key phenological stages. The SPAD index was measured at R_2_ stage, using a chlorophyl LOG device (Falcon^®^, Falcon Instruments, Boulder, CO, USA), by assessing the central third of each leaflet from the third fully expanded trifoliate leaf. Leaf area per plant was determined at the R_3_ stage by measuring the length and width of each leaflet in all replicates and multiplying the product by 0.7 [46]. Stem diameter was measured at three positions along the basal portion of the stem (5, 10, and 15 cm above the soil surface) also at the R_3_ stage. The average of the three values was calculated for each replicate and used as the representative stem diameter.

Leaf temperature was recorded using a handheld laser infrared thermometer. Measurements were taken in the central region of the leaflet of the third trifoliate leaf, with all readings conducted at the R_3_ stage under uniform conditions across replicates

Reproductive parameters, including the number of pods per plant, number of grains per plant, and grain dry mass per plant, were recorded at physiological maturity. A subsample of the harvested grains was dried in a forced-air oven at 60 °C for 48 h to determine dry weight and adjust for moisture content.

Grain nitrogen content was analyzed using the total N method following sulfuric acid digestion in a digestion block. Protein content was calculated by multiplying the grain N concentration (on a dry basis) by a factor of 6.25 [47]. Total protein accumulation per plant was determined by multiplying the grain protein content by the grain dry mass per plant.

### 4.4. Experiment II Measurements

Leaf area and SPAD index in Experiment II were assessed using the same methodology in Experiment I. Relative water content (RWC) was measured at the R_2_ stage, with samples collected at 09:00 a.m. From each plant, ten leaf discs (1.5 mm radius) were excised from the third fully expanded trifoliolate leaf. Fresh mass (Mf) was recorded immediately after collection. The discs were then immersed in deionized water for 6 h to obtain turgid mass (Mt), followed by oven-drying at 60 °C until constant weight to determine the dry mass (Md). RWC was calculated using the formula proposed by Barrs & Weatherley (1962) [48]:

$$RWC\;\left(\%\right)=\frac{Mf-Md}{Mt-Md}\times 100$$

Electrolyte leakage (EL) was determined at the R_3_ stage, following the method described by Blum et al. [49]. Ten leaf discs (1.5 mm radius) were collected from the third trifoliate leaf (counting from the base of the plant) and placed in a beaker containing 20 mL of deionized water. After 2 h of incubation at room temperature, the initial electrical conductivity (Ci) of the solution was measured using a benchtop conductivity meter (TEC-4MP, Tecnal^®^ Equipamentos Científicos Ltd.a., Piracicaba, SP, Brazil). The samples were then incubated in an oven at 65 °C for 3 h, after which the final electrical conductivity (Cf) was recorded. Electrolyte leakage was calculated using the following equation:

$$EL=\;\frac{Ci}{Cf}\times 100\;$$

At the R_3_ stage chlorophyll *a*, *b*, total chlorophyll, and carotenoids contents were determined. Five leaf discs (1.5 mm radius) were collected from the third fully expanded trifoliolate leaf and placed into aluminum-coated Eppendorf tubes containing 80% acetone. Samples were incubated in the dark at 4 °C for 72 h to allow complete pigment extraction. Absorbance was measured using a fluorescence spectrometer (BEL1105, Tecnal^®^ Equipamentos Científicos Ltda., Piracicaba, SP, Brazil) following the protocol of Lichtenthaler (1987) [50].

At the R_5_ stage (grain filling), physiological metabolites were analyzed. Samples were collected at 07:00 h a.m. from the third trifoliate leaf and immediately frozen in liquid nitrogen in situ, then stored at −80 °C until analysis. Ascorbic acid content was determined according to the method of [51], and total phenolic compounds were quantified following [52]. The concentrations of isoflavonoids (daidzein, daidzin, genistein and genistin) were determined using ultra-performance liquid chromatography UPLC, following protocols described by [53,54] for isoflavonoids and [54,55] for amino acids. Results were expressed in mg 100 g^−1^ of dry matter.

Foliar Si content was also assessed at the R_5_ stage. Leaves were collected from the third trifoliolate leaf (counting from the base of the plant), washed sequentially with tap water, a 0.1% detergent solution, 3% HCl, and finally with deionized water. Samples were dried in a forced-air oven at 65 ± 5 °C), then ground into a fine powder [56]. Dry digestion method was used to determine the Si concentration, following the method described by Elliott et al. 1991 [57].

At the R_6_ stage (full seed), a descriptive analysis of root morphological attributes was conducted. Intact root systems were separated by gently washing the soil substrate in water to minimize mechanical damage. Root analysis followed the protocol outlined by Bouma et al. (2000) [58]. The washed roots were carefully arranged in an acrylic tray filled with water to ensure full submersion and scanned at a resolution of 300 dpi using an Epson XL 10000 flatbed scanner (Epson America, Inc., Long Beach, CA, USA). Quantitative analysis of root traits was performed using WinRhizo software, version 4.1c (Regent Instruments Inc., Québec City, QC, Canada, 2017). The following parameters were assessed: root volume, total length, projected area and biomass. After scanning, the roots were dried in a forced-air oven and weighed using an analytical balance to determine dry root biomass. In addition, soil samples were collected from each treatment at the end of the experiment. The available Si content in the soil was quantified using the method proposed by van Raij method (2001) [39].

### 4.5. Statistical Analysis

Data were tested for homogeneity of variances using Levene’s test and for normality using the Shapiro–Wilk test. When both assumptions were made, treatment means were compared by Tukey’s Honest Significant Difference (HSD) test at a significance level of α = 0.05, considering the interaction between factors in a two-way factorial design. If either assumption was violated, the nonparametric Kruskal–Wallis test was applied independently for each factor level (α = 0.05). The principal component analysis (PCA) was conducted using the “FactoMineR” package in combination with “ggplot2”, including only numerical variables, with 95% confidence ellipse applied to visualize group separation. Prior to PCA, all variables were standardized (mean-centered and scaled to unit variance) to account for differences in measurement scales. All statistical analyses were performed in RStudio environment, version 2024.12.0 + 467 (RStudio, PBC, Boston, MA, USA).

## 5. Conclusions

The use of sorbitol-stabilized Si source (SiKe), was an effective strategy for enhance Si uptake and improving soybean performance under both drought stress and well-watered conditions. Across two complementary experiments, SiKe consistently promoted root development, increased biomass accumulation, enhanced antioxidant capacity, and modulated key primary and secondary metabolic pathways, particularly isoflavonoid biosynthesis, thereby improving tolerance to oxidative stress. These coordinated responses suggest that SiKe strengthens physiological resilience while maintaining growth and metabolic homeostasis, especially under water deficit conditions.

Under well-watered conditions, Si application supported greater pod set, while under drought stress, it sustained root development and preserved physiological function. This dual functionality highlights Si’s multifaceted role in both stress mitigation and growth promotion. Collectively, the findings reinforce Si’s potential as a resilience-enhancing element and a valuable tool for promoting crop stability in the face of climatic variability, contributing to more sustainable agricultural systems.

Future research should focus on evaluating stabilized Si formulations under diverse soil and climatic field conditions, investigating the temporal dynamics of foliar Si absorption and internal redistribution, and elucidating the molecular pathways modulated by SiKe, particularly those associated with phenylpropanoid metabolism and osmotic adjustment. Additionally, identifying the most effective phenological stages for Si application will be essential to optimizing its benefits. Advancing these research fronts will help establish stabilized Si as a practical and scalable solution for enhancing soybean resilience and productivity in an era of increasing environmental stress.

## Figures and Tables

**Table 1:** Chemical analysis of the soil used in the experiment.

pH CaCl_2_	O.M.	P (resin)	S	Ca	Mg	K	Al	H + Al	BS	CEC	BS	m
	g dm^−3^	mg dm^−3^		mmol_c_ dm^−3^							%	%
5.71	22.9	17.6	10.2	37.6	12	3.24	<0.1	14.1	52.8	66.9	79	0
B	Cu	Fe	Mn	Zn	Na	Si	Sand total	Silt	Clay	Texture class		
mg dm^−3^							g kg^−1^					
0.4	1.1	25.3	38.2	2.9	9	<9.7	751	25	224	Medium		

O.M., organic matter; P (resin), phosphorus (resin); S, sulphur (Ca_3_(PO_4_)_2_ 0,01 mol L^−1^); Ca, calcium (KCl 1 mol L^−1^); Mg, magnesium (KCl 1 mol L^−1^); K, potassium (resin); Al, aluminium (KCl 1 mol L^−1^); H + Al, (SMP), BS, base saturation; CEC, cation exchange capacity; m, Al saturation; B, boron (hot water); Cu, cupper (DTPA); Fe, iron (DTPA); Mn, manganese (DTPA); Zn, zinc (DTPA); Na (Mehlich1); Si, silicon (CaCl_2_ 0.01 mol L^−1^) [39].

## Data Availability

During the preparation of this work the authors used ChatGPT^®^ (OpenAI), based on the GPT-5.2 language model in order to check scientific writing English style. After using this tool, the authors reviewed and edited the content as needed and take full responsibility for the content of the publication.

## Supplementary Materials

The following supporting information can be downloaded at: https://www.mdpi.com/article/10.3390/plants15020197/s1, Table S1. Schedule of the water deficit stress period in Experiment I.; Table S2. Schedule of the water deficit stress period in Experiment II.; Table S3. *p*-value and CV (%) of each variable of the experiments.; Table S4. Loadings of the variables on the principal components (PC1 and PC2) obtained from the principal component analysis (PCA). Positive and negative values indicate the direction and magnitude of each variable’s contribution to each component.

## Abbreviations

The following abbreviations are used in this manuscript:

SiK	Potassium silicate (K_2_SiO_3_)
SiKe	Potassium silicate (K_2_SiO_3_) + Sorbitol (C_6_H_14_O_6_)
ROS	Reactive oxygen species
RSN	Reactive nitrogen species
^1^O_2_	Singleton Oxygen
O_2_^−^	Superoxide
Vn	Soybean’s vegetative stages
Rn	Soybean’s reproductive stages
SPAD	Soil–Plant Analysis Development
EL	Electrolyte leakage
Ci	Initial conductivity
Cf	Final conductivity
DM	Dry matter
UV-B	Ultraviolet-B radiation
PCA	Principal component analysis
