# Supplementary material for: Sorbitol-Stabilized Silicon Formulation Improve Root Traits and Antioxidant Response in Drought-Stressed Soybean
Source: Plants (Basel). 2026 Jan 8;15(2):197. doi: 10.3390/plants15020197 (PMC12845457; doi:10.3390/plants15020197)
Supplement: Supplementary file 1 [file plants-15-00197-s001.zip › plants-4012966-SI.pdf]

**Table S1.** Schedule of the water deficit stress period in Experiment I.

| November, 2023 | | | | | | | December, 2023 | | | | | | | January, 2024 | | | | | | |
|---|---|---|---|---|---|---|---|---|---|---|---|---|---|---|---|---|---|---|---|---|
| Sun. | Mon. | Tue. | Wed. | Thu. | Fri. | Sat. | Sun. | Mon. | Tue. | Wed. | Thu. | Fri. | Sat. | Sun. | Mon. | Tue. | Wed. | Thu. | Fri. | Sat. |
| | | | 1 | 2 | 3 | 4 | | | | | | 1 | 2 | | 1 | 2 | 3 | 4 | 5 | 6 |
| 5 | 6 | 7 | 8 | 9 | 10 | 11 | 3 | 4 | 5 | 6 | 7 | 8 | 9 | 7 | 8 | 9 | 10 | 11 | 12 | 13 |
| 12 | 13 | 14 | 15 | 16 | 17 | 18 | 10 | 11 | 12 | 13 | 14 | 15 | 16 | 14 | 15 | 16 | 17 | 18 | 19 | 20 |
| 19 | 20 | 21 | 22 | 23 | 24 | 25 | 17 | 18 | 19 | 20 | 21 | 22 | 23 | 21 | 22 | 23 | 24 | 25 | 26 | 27 |
| 26 | 27 | 28 | 29 | 30 | | | 24 | 25 | 26 | 27 | 28 | 29 | 30 | 28 | 29 | 30 | 31 | | | |
| | | | | | | | 31 | | | | | | | | | | | | | |
| February, 2024 | | | | | | | March, 2024 | | | | | | | April, 2024 | | | | | | |
| Sun. | Mon. | Tue. | Wed. | Thu. | Fri. | Sat. | Sun. | Mon. | Tue. | Wed. | Thu. | Fri. | Sat. | Sun. | Mon. | Tue. | Wed. | Thu. | Fri. | Sat. |
| | | | | 1 | 2 | 3 | | | | | | 1 | 2 | | 1 | 2 | 3 | 4 | 5 | 6 |
| 4 | 5 | 6 | 7 | 8 | 9 | 10 | 3 | 4 | 5 | 6 | 7 | 8 | 9 | 7 | 8 | 9 | 10 | 11 | 12 | 13 |
| 11 | 12 | 13 | 14 | 15 | 16 | 17 | 10 | 11 | 12 | 13 | 14 | 15 | 16 | 14 | 15 | 16 | 17 | 18 | 19 | 20 |
| 18 | 19 | 20 | 21 | 22 | 23 | 24 | 17 | 18 | 19 | 20 | 21 | 22 | 23 | 21 | 22 | 23 | 24 | 25 | 26 | 27 |
| 25 | 26 | 27 | 28 | 29 | | | 24 | 25 | 26 | 27 | 28 | 29 | 30 | 28 | 29 | 30 | | | | |
| | | | | | | | 31 | | | | | | | | | | | | | |
| | Sowing | | | | | | | | | | | | | | | | | | | |
| | Foliar applications | | | | | | | | | | | | | | | | | | | |
| | Stress periods | | | | | | | | | | | | | | | | | | | |
| | SPAD evaluations | | | | | | | | | | | | | | | | | | | |
| | Foliar temperature evaluations | | | | | | | | | | | | | | | | | | | |
| | Stem diameter and leaf area | | | | | | | | | | | | | | | | | | | |
| | Yield evaluation | | | | | | | | | | | | | | | | | | | |

**Table S2.** Schedule of the water deficit stress period in Experiment II.

| December, 2023 | | | | | | | January, 2024 | | | | | | | February, 2024 | | | | | | |
|---|---|---|---|---|---|---|---|---|---|---|---|---|---|---|---|---|---|---|---|---|
| Sun. | Mon. | Tue. | Wed. | Thu. | Fri. | Sat. | Sun. | Mon. | Tue. | Wed. | Thu. | Fri. | Sat. | Sun. | Mon. | Tue. | Wed. | Thu. | Fri. | Sat. |
| | | | | | 1 | 2 | | 1 | 2 | 3 | 4 | 5 | 6 | | | | | 1 | 2 | 3 |
| 3 | 4 | 5 | 6 | 7 | 8 | 9 | 7 | 8 | 9 | 10 | 11 | 12 | 13 | 4 | 5 | 6 | 7 | 8 | 9 | 10 |
| 10 | 11 | 12 | 13 | 14 | 15 | 16 | 14 | 15 | 16 | 17 | 18 | 19 | 20 | 11 | 12 | 13 | 14 | 15 | 16 | 17 |
| 17 | 18 | 19 | 20 | 21 | 22 | 23 | 21 | 22 | 23 | 24 | 25 | 26 | 27 | 18 | 19 | 20 | 21 | 22 | 23 | 24 |
| 24 | 25 | 26 | 27 | 28 | 29 | 30 | 28 | 29 | 30 | 31 | | | | 25 | 26 | 27 | 28 | 29 | | |
| 31 | | | | | | | | | | | | | | | | | | | | |
| March, 2024 | | | | | | | April, 2024 | | | | | | | May, 2024 | | | | | | |
| Sun. | Mon. | Tue. | Wed. | Thu. | Fri. | Sat. | Sun. | Mon. | Tue. | Wed. | Thu. | Fri. | Sat. | Sun. | Mon. | Tue. | Wed. | Thu. | Fri. | Sat. |
| | | | | | 1 | 2 | | 1 | 2 | 3 | 4 | 5 | 6 | | | | 1 | 2 | 3 | 4 |
| 3 | 4 | 5 | 6 | 7 | 8 | 9 | 7 | 8 | 9 | 10 | 11 | 12 | 13 | 5 | 6 | 7 | 8 | 9 | 10 | 11 |
| 10 | 11 | 12 | 13 | 14 | 15 | 16 | 14 | 15 | 16 | 17 | 18 | 19 | 20 | 12 | 13 | 14 | 15 | 16 | 17 | 18 |
| 17 | 18 | 19 | 20 | 21 | 22 | 23 | 21 | 22 | 23 | 24 | 25 | 26 | 27 | 19 | 20 | 21 | 22 | 23 | 24 | 25 |
| 24 | 25 | 26 | 27 | 28 | 29 | 30 | 28 | 29 | 30 | | | | | 26 | 27 | 28 | 29 | 30 | 31 | |
| 31 | | | | | | | | | | | | | | | | | | | | |
| | Sowing | | | | | | | | | | | | | | | | | | | |
| | Foliar applications | | | | | | | | | | | | | | | | | | | |
| | Stress periods | | | | | | | | | | | | | | | | | | | |
| | SPAD evaluations | | | | | | | | | | | | | | | | | | | |
| | Phyisiological measurements | | | | | | | | | | | | | | | | | | | |
| | Root analyse | | | | | | | | | | | | | | | | | | | |

**Table S3.** *p*-value and CV (%) of each variable of the experiments.

| | **Water condition** | **Si** | **Water condition x Si** | **CV (%)** |
|---|---|---|---|---|
| SPAD index | <0.0001*** | <0.0001*** | 0.0189NS | 7.4 |
| Leaf area ($cm^2$) (I) | 0.1126NS | 0.0032** | 0.1134NS | 14.25 |
| Steam diameter (cm) | <0.0001*** | 0.2539 | 0.1198NS | 9.29 |
| Leaf temperature (°C) | <0.0001*** | 0.6022NS | 0.5671NS | 6.57 |
| Pods per plant (no.) | <0.0001*** | 0.0223* | 0.0448* | 16.88 |
| Grains per plant (no.) | <0.0001*** | 0.4062NS | 0.8299NS | 19.98 |
| 1000-grain weight | 0.0073** | 0.9133NS | 0.1095NS | 14 |
| Grain protein content (g $kg^{-1}$) | 0.2638NS | 0.7832NS | 0.7144NS | 9.83 |
| Si content in the leaf | <0.0001*** | <0.0001*** | 0.8934NS | 9.7 |
| Root length (cm) | <0.0001*** | <0.0001*** | 0.0071** | 17.5 |
| Root volume ($cm^3$) | <0.0001*** | <0.0001*** | 0.1009NS | 22.75 |
| Root projected area ($cm^2$) | <0.0001*** | <0.0001*** | 0.0258* | 19.29 |
| Root biomass (kg) | <0.0001*** | <0.0001*** | 0.8357NS | 21.88 |
| Leaf area ($cm^2$) (II) | 0.1888NS | 0.1924NS | 0.6942NS | 12.98 |
| Water content in the leaf (%) | <0.0001*** | 0.4324NS | 0.7158NS | 10.94 |
| Electrolyte leakage (mS $cm^{-1}$) | 0.0029** | 0.2986NS | 0.9972NS | 15.08 |
| Total chlorophyll (μg $ml^{-1}$) | 0.0983NS | 0.2459NS | 0.4646NS | 47.62 |
| Carotenoids (μg $ml^{-1}$) | 0.0552NS | 0.1515NS | 0.3867NS | 44.69 |
| SPAD index (II) | 0.0789NS | 0.0874NS | 0.0844NS | 11.03 |
| Ascorbic acid (μg $ml^{-1}$) | <0.0001*** | <0.0001*** | <0.0001*** | 12.18 |
| Total pheophytin (μg $ml^{-1}$) | 0.1048NS | 0.1777NS | 0.4256NS | 45.95 |
| Total Isoflavonoids (mg $100g^{-1}$) | 0.2175NS | 0.0001*** | <0.0001*** | 26.35 |
| Daidzein (mg $100g^{-1}$) | 0.0264* | <0.0001*** | 0.9692NS | 70.39 |
| Daidzin (mg $100g^{-1}$) | 0.3871NS | 0.0003** | <0.0001*** | 47.77 |
| Genistein (mg $100g^{-1}$) | 0.2716NS | 0.8631NS | 0.2887NS | 121.06 |
| Genistin (mg $100g^{-1}$) | 0.1143NS | <0.0001*** | 0.001** | 36.74 |
| Aspartic Acid (mg $100g^{-1}$) | 0.2307NS | 0.5037NS | 0.7743NS | 56.05 |
| Alanine (mg $100g^{-1}$) | 0.8567NS | 0.1302NS | 0.1398NS | 88.7 |
| Cysteine (mg $100g^{-1}$) | 0.356NS | 0.954NS | 0.631NS | 73 |
| Phenylalanine (mg $100g^{-1}$) | 0.622NS | 0.825NS | 0.173NS | 92.6 |
| Histidine (mg $100g^{-1}$) | 0.308NS | 0.0458* | 0.0617NS | 82.15 |
| Lysine (mg $100g^{-1}$) | 0.6403NS | <0.0001*** | 0.2883NS | 71.4 |
| Proline (mg $100g^{-1}$) | 0.7572NS | 0.0487* | 0.2018NS | 98.3 |
| Serine (mg $100g^{-1}$) | <0.0001*** | <0.0001*** | <0.0001*** | 112.38 |
| Tyrosine (mg $100g^{-1}$) | 0.9609NS | 0.147NS | 0.7156NS | 33.52 |
| Threonine (mg $100g^{-1}$) | 0.2867NS | 0.0039** | 0.099NS | 66.67 |
| Valine (mg $100g^{-1}$) | 0.2406NS | 0.0531NS | 0.436NS | 37.25 |
| Total amino acids (mg $100g^{-1}$) | 0.7996NS | 0.0391* | 0.4867NS | 34.99 |

NS, non-significant; ***, significant at $p < 0.001$; **, significant at $p < 0.01$; *, significant at $p < 0.05$.

**Table S4.** Loadings of the variables on the principal components (PC1 and PC2) obtained from the principal component analysis (PCA). Positive and negative values indicate the direction and magnitude of each variable's contribution to each component.

| Variables | PC1 | PC2 |
|---|---|---|
| Si concentration in the leaf | 0.2749 | 0.0533 |
| Leaf area | 0.0280 | -0.2876 |
| Steam diameter | 0.2253 | 0.1237 |
| Root biomass | 0.2610 | -0.0972 |
| Root length | 0.2653 | -0.1328 |
| Root volume | 0.2762 | -0.0680 |
| Root projected area | 0.2693 | -0.0942 |
| Relative water content | 0.2355 | 0.1784 |
| Electrolyte leakage | -0.1414 | -0.2657 |
| Chlorophyll A | 0.1350 | -0.0447 |
| Chlorophyll B | 0.1958 | -0.1079 |
| Total chlorophyll | 0.2016 | -0.0665 |
| SPAD index | 0.1784 | 0.1841 |
| Carotenoids | 0.2024 | -0.1087 |
| Phenolic content | 0.0048 | 0.3688 |
| Ascorbic acid | 0.2401 | -0.2231 |
| Leaf temperature | -0.1818 | -0.2221 |
| Pods per plant | 0.2813 | 0.1712 |
| Grains per plant | 0.1973 | 0.2170 |
| Grain protein content | -0.0608 | -0.1955 |
| Total mass of protein produced per plant | 0.1915 | 0.2436 |
| Daidzein | 0.1764 | -0.3145 |
| Daidzin | -0.0610 | 0.2384 |
| Genistein | 0.0973 | 0.1777 |
| Genistin | -0.1770 | 0.2052 |
| Total Isoflavonoids | -0.1236 | 0.2321 |
